# Polymorphisms of the PRLR Gene and Their Association with Milk Production Traits in Egyptian Buffaloes

**DOI:** 10.3390/ani11051237

**Published:** 2021-04-25

**Authors:** Mohammed A. El-Magd, Aziza Fathy, Khaled A. Kahilo, Ayman A. Saleh, Ahmed I. El Sheikh, Salah AL-Shami, Shymaa M. El-Komy

**Affiliations:** 1 Department of Anatomy & Embryology, Faculty of Veterinary Medicine, Kafrelsheikh University, Kafrelsheikh 33516, Egypt; 2 Department of Biochemistry, Faculty of Veterinary Medicine, Kafrelsheikh University, Kafrelsheikh 33516, Egypt; Doctor_rose10@yahoo.com (A.F.); genecom3@yahoo.com (K.A.K.); 3 Department of Animal Wealth Development, Veterinary Genetics & Genetic Engineering, Faculty of Veterinary Medicine, Zagazig University, Zagazig 44519, Egypt; Lateefsaleh@yahoo.com; 4 Department of Public Health, Faculty of Veterinary Medicine, King Faisal University, Alahssa 31982, Saudi Arabia; aelsheikh@kfu.edu.sa (A.I.E.S.); Salshami@kfu.edu.sa (S.A.-S.); 5 Department of Animal Wealth Development, Faculty of Veterinary Medicine, Alexandria University, Alexandria 21561, Egypt; 6 Department of Animal Production, Faculty of Agriculture, Tanta University, Tanta 31527, Egypt; dr_shymaelkomy@outlook.com

**Keywords:** prolactin receptor, mutations, Egyptian buffalo, milk performance

## Abstract

**Simple Summary:**

The two non-synonymous g.11685G>A and g.11773T>C SNPs of PRLR(L2) were significantly associated with milk yield, fat%, and protein%, and mRNA and protein levels of PRL and PRLR in milk somatic cells. GT-animals had the best milk performance; however, AC-animals had inferior milk production. Thus, the selection of buffaloes with GT haplotypes may enhance milk performance in Egyptian buffaloes.

**Abstract:**

Prolactin (PRL) and its receptor (PRLR) were considered as potential genetic markers for milk production and quality traits in cattle. However, little information is available regarding *PRLR* genetic diversity and association studies with milk traits in Egyptian water buffaloes. Therefore, the present study was conducted to search for mutations in *PRLR* and determine their associations with milk performance in these animals. Exon3 (E3) and E10 of *PRLR* were screened for polymorphisms using single strand conformation polymorphism (SSCP) and sequencing in 400 buffaloes. The associations between haplotypes and milk production (fat%, protein%, lactose%, and solid%) traits as well as mRNA and protein levels of PRL and PRLR were studied. Two single nucleotide polymorphisms (SNPs) in E10 were detected: g.11685G>A (p.Ala494Thr) and g.11773T>C (p.Val523Aal). The G and T alleles were wild (ancestral) alleles, while the A and C alleles were mutant alleles. These SNPs resulted in four haplotypes; AC, AT, GC, and GT. Buffaloes with wild GT haplotypes showed significantly higher milk yield, fat% and protein%, mRNA and protein levels of PRL and PRLR in milk somatic cells than other animals. Animals carrying mutant AC haplotype had inferior milk traits and lowest levels of associated mRNAs and proteins. With these results, we could conclude that the selection of buffaloes with wild GT haplotypes for g.11685G>A and g.11773T>C SNPs of the *PRLR* gene might improve the milk production traits of Egyptian water buffaloes.

## 1. Introduction

High milk production is one of the world’s most important priorities for dairy breeding. Milk composition characteristics are new breeding goals to cope with healthier dietary demands [1]. The selection of dairy animals with superior milk performance is of a great importance to breeders and consumers. Prolactin (PRL) is necessary for lactation [2] and this lactotrophic potential is facilitated by combining with its receptor, PRLR. During lactation, PRL/PRLR signaling not only stimulates the abundant synthesis of milk protein, lactose, and fat, but it also regulates their secretion [3], hence the inhibition of this signaling reduces milk yield [4].

PRLR belongs to the transmembrane cytokine class-1 receptor superfamily. The *PRLR* gene was mapped to chromosome 19 (buffalo), 20 (cattle), and 16 (sheep), and composed of 10 exons, of which exon1 (E1) and E2 are non-coding [5,6]. Similar to other bovine species, buffalo PRLR protein consists of four domains: signal peptide (24 amino acids encoded by E3 and E4); extracellular domain (encoded by E4-E7); transmembrane domain (encoded by E7 and E8 and contains PRL binding site); and intracellular (cytoplasmic) domain (encoded by E9 and E10) [7]. In bovine species, PRLR has two functionally different isoforms, short and long isoforms, produced mainly by alternative splicing [8], with a length of 296 and 581 amino acids, respectively [7]. The long isoform is responsible for almost all functions mediated by binding with PRL and plays a crucial role in the regulation of milk protein genes’ transcription through binding to certain transcription sites at their promoters [2]. However, the short isoform has less distinct function due to the absence of E10 [5].

Single strand conformation polymorphism (SSCP) and sequencing followed by statistical association analysis were successfully used to detect single nucleotides polymorphisms (SNPs) associated with production, fertility, growth, and milk traits in animals [9,10,11,12,13,14]. In ruminants, the majority of *PRLR* SNPs were detected in E3 and E10. Previous studies revealed significant associations between these SNPs and milk production traits in cattle [5,6,15,16], goat [17,18,19], and sheep [20].

Three previous studies screened partial sequences (E3, E7, E10) of *PRLR* for polymorphisms in Indian and Chinese buffalo breeds [21,22,23]. However, those studies used different nomenclature for the detected SNPs, probably due to the absence of wide genomic analysis. Recently, Cosenza et al. (7) screened a larger sequence (from E3 to 3’ UTR) for polymorphisms and found several SNPs. However, they only studied the association of one selected SNP in E10 (g.11188A>G) with milk fat quality and content in Italian river buffalo and found that milk of AA-genotype animals had higher contents of odd branched-chain fatty acids. Therefore, we used the most recent published sequence of Italian buffalo *PRLR* (GenBank accession number MF461277.1) as a reference for buffalo *PRLR* sequence [7] and accordingly only the names (but not the locations) of the prior detected SNPs were changed. Based on this nomenclature, two different studies detected g.114T>G SNP in E3 and g.11188A>G SNP in E10 of *PRLR* in Indian and Italian buffaloes [7,22]. Another non-synonymous g.11488A>G SNP was detected in E10 of Indian, but not Italian, river buffaloes [7,23]. Interestingly, a trans-specific silent mutation (g.11936G>A) was found in *PRLR* E10 of buffalo [7] and cattle [24]. Another seven mutations were determined in Italian buffalo *PRLR* E10, including four missense mutations (g.11434C>T, g.11577G>A, g.11580A>C, and g.11683C>T), and three silent mutations (g.11687A>G, g.11768T>C, and g.11882G>A) [7]; however, their associations with animals phenotypic traits have not been investigated yet.

As described above, some *PRLR* polymorphisms have been found in Indian, Chinese, and Italian buffaloes and their association with milk fat in Italian buffalo breeds has been examined [7,19,21,22,23]. However, to date, none of the detected SNPs have been explored in Egyptian water buffaloes. Moreover, these previous studies did not investigate associations between SNPs and the gene and protein expression of PRL or PRLR. Given that most detected SNPs were found in E3 and E10 of *PRLR*, herein we screened these two exons for polymorphisms and studied their associations with milk performance and the expression of PRL and PRLR.

## 2. Materials and Methods

Before conducting this study, we received ethical approval from the Animal Ethical Committee of Kafrelsheikh University with a license number of KFS 127/14.

### 2.1. Animals and Sampling

All animals (n = 400) enrolled in this study were randomly chosen from one large buffalo station in Kafrelsheikh province. The selected animals were daughters of 72 sires with 2 to 30 daughters per sire. Animals were milked twice daily with an equal interval of 12 h. Milk yield per 305-day lactation was obtained from farm records (from February 2014 till December 2018). Milk samples (n = 10,130 samples, from 1224 lactations) were collected from the 400 buffaloes. Milk constituents of protein, lactose, fat, and total solid (expressed in percentages) were determined by a milk analyzer (Funke Gerber, Berlin, Germany).

For the detection of polymorphisms, blood samples were obtained from veins of the 400 animals (5 mL/animal). For the extraction of mRNA and protein, milk samples (n = 9/haplotype) were gathered from animals with similar lactation age and stage. Milk somatic cell pellets were prepared by double centrifugations (1500× *g*/30 min followed by 1100× *g/*15 min) of these milk samples (1 L milk/animal). Milk somatic cells (SCs) are a group of cells exfoliated mainly from mammary gland epithelial cells (GECs) in addition to some WBCs and micro-organisms. Unlike GECs, SCs are easily extracted from milk samples in forms of pellets. They were also effectively utilized to assess the expression of some milk performance-related genes and proteins in buffalo and goat [14,25,26].

### 2.2. Polymerase Chain Reaction (PCR)

Genomic DNA was extracted from blood samples using GeneJET genomic DNA extraction kit following the manufacturer protocol (Thermo Scientific, #K0721, EU). Two loci of *PRLR* gene encompassing E3 [*PRLR*(L1)] and E10 [*PRLR*(L2)] were amplified by PCR using specific primers designed based on the published buffalo sequences in GenBank databases (Table 1). PCR mixture and conditions were performed as previously detailed [10]. The only difference was the annealing temperature which was set at 56 °C for 40 s in the present study. Agarose gels (1%) were used to confirm amplicon sizes.

### 2.3. SNP Identification by Single-strand Conformation Polymorphism and Sequencing

Single-strand conformation polymorphism (SSCP) was performed for all PCR products as previously described [12,13]. Animal genotypes were identified according to PCR-SSCP banding patterns. Sixty random purified PCR products: 50 from *PRLR*(L2) (10 from each SSCP banding pattern) and 10 from *PRLR*(L1) were sequenced by outsourcing (Macrogen Company, South Korea) and the obtained sequences were analyzed by Geneious 4.8.4 software (Biomatters, Ltd, Auckland, New Zealand). PROMO software was used to predict transcription factor binding sites results from nucleotides substitutions on *PRLR*(L1) relative to Italian and Indian buffaloes [27].

### 2.4. Real-Time PCR

The commercially available kit GeneJET RNA (Thermo Scientific, # K0731, Waltham, MA, USA) was used to extract RNA from milk somatic cell pellets as previously detailed [14]. Purity and concentration of RNA samples were assessed by Nanodrop (Q5000, Quawell, San Jose, CA, USA). The qRT-PCR mixture contained cDNA, primers of candidate genes (Table 1), and SYBR Green Master Mix (Thermo Scientific, # K0221, Waltham, MA, USA). Each sample was run in triplicate along with nontemplate control in each plate. The thermal cycling conditions and determination of gene expression (expressed as fold change against the internal control β-actin) were performed as previously detailed [28,29]. Expression profiles of *PRL* and *PRLR* in milk SCs were validated by detecting their expression in mammary gland tissues as previously described [14].

### 2.5. Western Blot

The expression of PRL and PRLR in milk SCs was determined by Western blot as previously described [14]. Full information (type, source, and dilution) about the used antibodies is presented in Table S1. β-actin protein was used as a housekeeping protein and protein bands were quantified by Image J software.

### 2.6. Statistical Analysis

Allele, haplotype, genotype, and minor allele frequencies, gene heterozygosity, effective allele numbers, polymorphism information content, Hardy–Weinberg equilibrium, and linkage disequilibrium were calculated as previously described [14]. Associations between *PRLR*(L2) haplotypes and milk yield and composition were analyzed using a mixed linear model by SAS V9 (SAS Inst. Inc., Cary, NC, USA) as previously described [14]. In brief, *y_ijklmn_* = μ + Sire*_i_* + A*_j_* + P*_k_* + L*_l_* + M*_m_* + H*_n_*_+_ e*_ijklmn_* where Y represents the value of milk yield and composition traits; μ is the overall mean for each trait; Sire*_i_* is the random effects of the *i*^th^ sires; A*_j_* is the fixed effect of the age of the *j*^th^ animal at calving expressed in years (6 levels: 1 = <4yrs., 2 = 4 yrs., 3 = 5 yrs., 4 = 6 yrs., 5 = 7 yrs., 6 = >7 yrs.); P*_k_* is the fixed effect of the parity (3 levels: parity 1, 2 and 3–5); L*_l_* is the fixed effect of the *l*^th^ stage of lactation (10 levels of 30 days each); M*_m_* is the fixed effect of the *m*^th^ month of calving (12 levels); H*_n_* is the fixed effect of the *n*^th^ *PRLR* haplotype with 4 levels (*n* = AC, AT, GC, and GT); and e*_ijklmn_* is the random residual effect.

Univariate analyses were used to test the association of the fixed effects and the dependent variables of interest setting a liberal p-value of (*p* < 0.25). Then, final significance for testing the fixed effects in the multivariable model was established at *p* < 0.05. The results of the multiple comparisons were corrected using Bonferroni correction, and the differences were considered significant at *p* < 0.05. Data were expressed as least squares means ± standard error of mean (SEM). Difference in expression levels of candidate genes and proteins among different haplotypes were plotted using GraphPad Prism 8 (GraphPad Software, Inc., San Diego, CA, USA).

## 3. Results and Discussion

### 3.1. Analysis of the Detected SNPs

**Figure 1:**
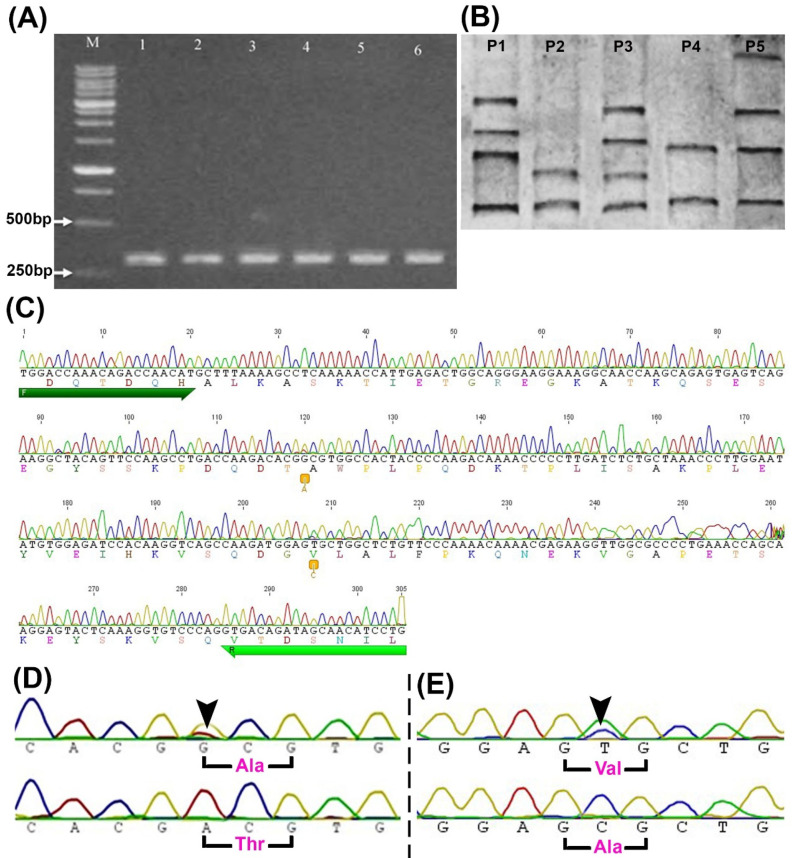
Identification of SNPs in buffalo *PRLR*(L2). (**A**) Agarose gel of *PRLR*(L2) amplified fragments (305 bp) from six different samples (lanes 1-6). (**B**) PCR-SSCP banding patterns show five different patterns (P1-P5) in five different samples. (**C**) A representative sequence chromatogram from one sample shows the sites of the two SNPs (two orange boxes), primers (forward (F) and reverse (R), the two green arrows), and amino acid sequences (colored letters). (**D**,**E**) Sequences chromatogram spanning the site of g.11685G>A (**D**) and g.11773T>C (**E**) SNPs (arrowheads) and the altered amino acids (p.Ala494Thr and p.Val523Ala). Ala, alanine; M; 1Kb ladder; Thr, threonine; Val, valine.

The *PRLR*(L1) (Figure S1) and *PRLR*(L2) (Figure 1A) were genotyped in all animals using PCR-SSCP. The *PRLR*(L2) showed five different SSCP banding patterns (P1-P5, Figure 1B), suggesting the presence of polymorphisms, while *PRLR*(L1) showed only one pattern (Figure S2), implying the absence of polymorphisms in this locus. Indeed, data obtained from sequencing revealed no polymorphisms in *PRLR*(L1) (Figure S3) among the Egyptian buffaloes. In contrast, analysis of *PRLR*(L2) sequences (which were submitted to GenBank with an accession number of JQ045623.1) revealed the presence of two non-synonymous SNPs; g.11685G>A (at nucleotide (nt) 625 of E10 that changed alanine to threonine amino acid (aa), (p.Ala494Thr)) (Figure 1C,D) and g.11773T>C (at nt 713 of E10 that changed valine to alanine aa, (p.Val523Aal) (Figure 1C,E). The sequences of the different 5 SCCP patterns are shown in the supplementary file (Figure S4).

Comparing nucleotide sequences of *PRLR*(L2) with the published sequences of various ruminant species (buffalo, cattle, sheep, goat, and camel) revealed the presence of g.11685G and g.11773T alleles in all ruminants. Therefore, these two alleles were considered as wild (ancestral) type alleles. However, the other two alleles (g.11685A and g.11773C) were found only in Egyptian buffalo sequences (except the C allele, which was also found in camel) and could be mutant alleles (Table S2). Among different buffalo breeds (Table 2), seven SNPs were detected at *PRLR*(L2): two unique SNPs (g.11685G>A and g.11773T>C) in Egyptian buffaloes (this study); three unique SNPs (g.11577G>A, g.11683C>T, and g.11768T>C) in Italian buffalo; and two shared SNPs (g.11580A>C and g.11687A>G) in Italian and Indian buffaloes [7,22,23]. Surprisingly, Egyptian buffaloes had only the C (mutant) allele of the shared (g.11580A>C) SNP, which could argue for the presence of the latter SNP if a large population of Egyptian buffaloes was examined. This higher level of nucleotide polymorphisms in *PRLR*(L2), as a part of E10, could be mainly due to alternative splicing characteristics for other regions of the *PRLR* as compared to E10 [8]. As compared to sequences of other ruminants (Table S2), none of the Egyptian buffaloes *PRLR*(L2) SNPs were found in other ruminant species studied thus far [5,18,20]. At the protein level, the wild p.Ala494 residue of g.11685G>A SNP was conserved in most studied species (Italian and Indian buffaloes, sheep and goat), while the mutant p.Thr494 residue was not detected in all studied animals thus far (Table S2). This high degree of Ala conservation implies that amino acid replacements at this codon may influence PRLR function. On the other hand, the wild p.Val523 residue of g.11773T>C SNP was conserved in Italian and Indian buffaloes, and cattle, while, interestingly, the buffalo mutant p.Aal523 residue was found in sheep, goat, and camel but as a wild residue.

A comparison between nucleotide sequences of *PRLR*(L1) and both Indian (accession no GQ339914) and Italian (accession no MF461277.1) river buffaloes showed an insertion of C nucleotide in the non-coding sequence of E3 in Egyptian buffalo *PRLR*(L1) (Figure S5). Mutation in this site of the promoter region could change the transcription factors binding sites (TFBS) that regulate gene expression. Therefore, we applied in silico prediction analysis for TFBS and found a new TFBS in *PRLR*(L1) for the cancer-suppressor *p53* gene due to this insertion, suggesting a possible effect on PRLR expression and function. In support, we previously found two SNPs in the noncoding sequences of E1 and E4 of *IGF1* and reported their association with growth traits in Egyptian buffaloes [30]. We also found g.1268G>T (p. Val19Phe) SNP among Egyptian and foreign buffaloes (Figure S5).

### 3.2. Analysis of Genotype Frequencies, Genetic Indices and LD

The genotyping of 400 buffalo showed higher frequencies of the wild alleles (g.11685G: 0.52 and g.11773T: 0.55) and their homozygous genotypes (g.11685GG: 0.32 and g.11773TT: 0.32) than the mutant alleles (g.11685A: 0.48 and g.11773C: 0.45) and their homozygous genotypes (g.11685AA: 0.28 and g.11773CC: 0.225) (Table 2). The heterozygous genotypes showed higher frequencies (g.11685GA: 0.40 and g.11773TC: 0.455) than the homozygous genotypes in both SNPs. The three genotypes of the g.11685G>A SNP deviated from HWE (*p* < 0.05), while those of the g.11773T>C SNP fit HWE (*p* > 0.05) (Table 2). Deviation of HWE indicates the presence of either natural or artificial selection for g.11685G>A SNP in Egyptian buffaloes.

Both g.11685G>A and g.11773T>C SNPs had closest MAF value (0.48 and 0.45, respectively), high *Ne* values (1.99 and 1.98, respectively), similar medium PIC value (0.37), and similar high heterozygosity (the difference between expected and observed *He* = 0.49), indicating higher mutation frequencies of these SNPs (Table 2). Expectedly, as the two SNPs were close to each other in the same locus, the pairwise LD analysis revealed a very strong linkage disequilibrium (D’ = 1) between the two SNPs (Table 2 and Figure S6), suggesting their coinheritance.

### 3.3. Association of PRLR Haplotypes with Milk Yield and Composition

The inheritance of haplotype is better than that of individual SNPs [31]. In the present study, g.11685G>A and g.11773T>C SNPs resulted in four different haplotypes with the following frequencies: AC (0.225), AT (0.230), GC (0.225), and GT (0.320). This indicates higher frequencies in animals with the two wild alleles (GT). Since these two SNPs were completely linked, the effect of their four haplotypes (AC, AT, GC, and GT), rather than individual SNPs, on milk production traits was studied (Table 3). The four haplotypes were significantly (*p* < 0.05) associated with milk yield, fat%, and protein%, but no significance association was found with lactose% and total solid%. The animals with wild GT alleles showed significantly higher milk yield and fat%, and protein% than the other haplotypes. Moreover, the AC-haplotype animals had significantly lower milk yield and milk quality than the GC-and AT-haplotype animals, suggesting that the mutant AC alleles could be non-beneficial alleles for the studied milk traits. Overall, the GT haplotype was a favorable one for higher milk performance. These interesting findings indicate that the two detected mutations, especially g.11685G>A SNP, could be unwanted mutations for the animals.

A similar association, but for another SNP in E10 of *PRLR* (g.11188A>G, p.His328Arg), with milk fat quality and content was reported in Italian river buffalo with AA-genotype animals. This SNP was significantly associated with higher contents of milk odd branched-chain fatty acids [7]. Another study also reported a significant association between other non-synonymous SNPs in E10 of bovine *PRLR* (g.9206G>A and g.9681C>T) and milk yield and fat %, with superior performance for animals with the GGCC haplotype [5]. Similarly, Zhang et al. (15) also reported a significant association between *PRLR* polymorphisms and milk yield and fat% in cattle. However, these two previous studies did not find a significant association with protein%. Moreover, two other non-synonymous SNPs in E3 of bovine *PRLR* (g.1267 G>A and g.1268 T>C) were significantly associated with protein% and fat% [15,16]. In pigs, a non-synonymous c.1528A>T SNP in the *PRLR* gene was significantly associated with fat % and lactose %, with a superior performance in AA-genotype animals [32].

### 3.4. Association of SNP Haplotypes with mRNA and Protein Levels

**Figure 2:**
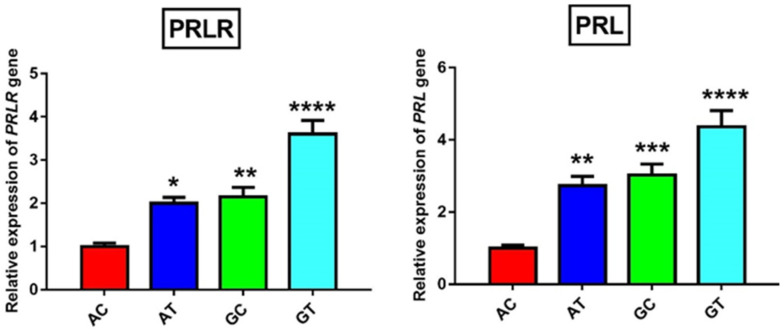
Real-time PCR graphical presentation showing expression of *PRLR* and *PRL* genes in milk SCs in animals carrying AC, AT, GC, and GT haplotypes. Data are expressed as fold change mean ± SEM. Number of samples per each haplotype was nine. The mutant AC haplotype was considered as the control. * *p* < 0.05, ** *p* < 0.01, *** *p* < 0.001, and **** *p* < 0.0001.

**Figure 3:**
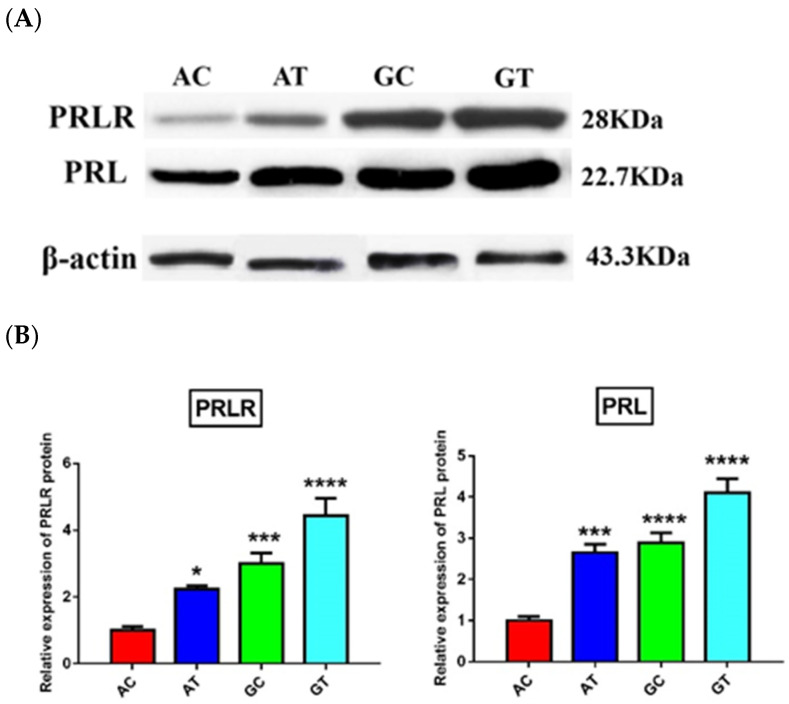
(**A**) Western blot bands showing expression of PRL and PRLR proteins in milk SCs in animals carrying AC, AT, GC, and GT haplotypes. (**B**) Band quantification of PRL and PRLR. Data are expressed as fold change mean ± SEM. Number of samples per each haplotype was nine. The mutant AC haplotype considered as the control. * *p* < 0.05, *** *p* < 0.001, and **** *p* < 0.0001.

One of the possible ways by which the non-synonymous SNPs of a gene could exert their influence on phenotypic traits is the alteration of gene expression and subsequently protein levels [33]. Both qRT-PCR and WB were used to detect mRNA/protein levels of PRL and PRLR in milk SCs. The obtained data revealed a significant (*p* ≤ 0.05) upregulation in the mRNA and protein levels of PRL and PRLR in SCs of animals carrying wild GT alleles as compared to the other three haplotypes (Figure 2 and Figure 3). Again, the mutant AC-haplotype animals exhibited significantly lower levels of PRL and PRLR mRNA and protein in SCs than heterogenous GC- and AT-haplotype animals. Collectively, animals carrying wild GT alleles showed superior milk performance accompanied by upregulated levels of PRL and PRLR mRNAs and proteins.

PRLR plays an essential role in lactation. PRLR upregulation is positively correlated with milk yield [34], while its inhibition reduces milk yield [4]. This could explain the association between higher milk yield and upregulated mRNA and protein levels of PRLR in GT-haplotype animals. Therefore, the question now is how SNPs in the *PRLR* gene would affect PRL protein expression. One of the probable answers is the disruption of the PRL-PRLR-JAK2/STAT signaling pathway by *PRLR* SNPs. PRL plays an important role in initiation and maintenance of lactation. PRL’s effect is mainly mediated through binding with the highly conserved extracellular domain of the PRLR, which further binds to and activates the intracytoplasmic targets of the JAK2/STAT signaling pathway [2], such as β-casein [35]. Although the two SNPs in E10 of the *PRLR* gene are located in the intracellular domain away from PRL binding site, it is likely that amino acid substitution as a result of these SNPs (p.Ala494Thr and p.Val523Aal) may change the functional structure of the PRLR intracellular domain in a way that inhibits its binding with JAK2/STAT downstream targets. Therefore, SNPs-induced downregulation of PRLR (similar to p.Ala494Thr and p.Val523Aal) could negatively affect PRL function and β-casein secretion. This notion could be further verified by proteins’ structural-functional interaction experiments. This also may explain why these two mutations are unfavorable for milk production traits.

The change in milk yield and protein% associated with g.11685G>A and g.11773T>C SNPs could be attributed to change in PRL and/or PRLR expression. However, the change of fat% could be indirect through linkage with other causative variants that might alter the expression of fat synthesis-related genes and proteins. PPARγ and DGAT1 induce the induction of triacylglycerol synthesis in GECs and thus they participate in milk fat synthesis and production [36,37]. To date, no study proves that PPARγ and DGAT1 could be downstream targets for the PRL-PRLR-JAK2/STAT signaling pathway. However, some clues indicate a close relationship between PPARγ and DGAT1, and the PRL-PRLR-JAK2/STAT pathway during lactogenesis and adipogenesis. Both PPARγ and PRLR are upregulated in GECs following the addition of PRL [19]. DGAT1 is downregulated in PPARγ-knockdown GECs and subsequently, the content of triacylglycerol decreases in the milk [36]. PRL can trigger upregulation of PPARγ expression in the multipotential mesenchymal stem cells NIH 3T3 [38]. PRLR-deficient preadipocytes lose their ability to express *PPARγ* [39]. Mice lacking PRLR have lower abdominal fat [40].

Although this study showed an association between two SNPs in *PRLR*(L2) and milk performance in buffaloes, we cannot assume that this is a causative polymorphism, as milk production is a complex trait which involves many genetic markers, loci, and quantitative trait loci (QTL). Therefore, further investigations using next-generation sequencing (NGS) to screen the whole genome in Egyptian buffaloes are required.

## 4. Conclusions

To the best of our knowledge, this is the first study to report the presence of two non-synonymous SNPs (g.11685G>A and g.11773T>C) in exon 10 of the *PRLR* gene of Egyptian buffaloes. These two SNPs resulted in four haplotypes (AC, AT, GC, and GT), which were significantly associated with milk yield, fat%, protein%, and expression of PRL and PRLR. The animals with wild GT haplotypes had better milk performance (higher milk yield, fat% and protein%) accompanied with higher expression of PRL and PRLR than animals with mutant AC haplotypes. Therefore, selection of GT-haplotype animals may improve milk production traits in Egyptian buffaloes.

## Figures and Tables

**Table 1:** Primers used in conventional and qRT-PCR.

Gene	Forward Primer	Reverse Primer	Ta (°C)	Localization *	Size (bp)	Experiment
PRLR(L1)	ATGTGCCTCACCAGACTTT	CCAGGGAGTGAAAAAGAAC	56	E3, partial introns2, 3	212	SNPs detection
PRLR(L2)	TGGACCAAACAGACCAACAT	CAGGATGTTGCTATCTGTCAC		E10 (g.11566–g.11870)	305	
PRLR	AACCATTGAGACTGGCAGGG	AAGGGGGTTTTGTCTTGGGG	60	E10	114	Relative expression by qRT-PCR
PRL	GCATGCTTGGCTCTAATGGG	TGTCAGTTTCTGCTATTTGTGAC		Coding sequences	186	
β-actin	CGACAACGGCTCCGGCATGT	CTCCTCAGGGGCCACACGGA			211	

* PRLR loci were determined based on the published Italian river buffalo sequence (accession no MF461277.1). The forward primer of PRLR(L1) precedes the starting nucleotide of MF461277.1 by 25 nt. Ta, annealing temperature. SNPs, single nucleotide polymorphisms.

**Table 2:** Genotypic and allelic frequencies, value of *χ*^2^ test, diversity parameter, and LD of g.11685G>A and g.11773T>C SNPs of buffalo *PRLR*(L2).

SNP	Genotype Frequency (Number)			Allele Frequency		χ2 (*p*-Value)	He	Ne	D’	MAF	PIC
g.11685G>A	GG	GA	AA	G	A	7.90 (0.019)	0.49	1.99	1.0		
	0.32 (128)	0.40 (160)	0.28 (112)	0.52	0.48					0.48	0.37
g.11773T>C	TT	TC	CC	T	C	1.42 (0.512)	0.49	1.98			
	0.32 (128)	0.455 (182)	0.225 (90)	0.55	0.45					0.45	0.37

D’, linkage disequilibrium (LD) coefficient; He, gene heterozygosity; MAF, minor allelic frequency, Ne, effective allele numbers; PIC, polymorphism information content; χ^2^, Chi-Square value.

**Table 3:** Association between *PRLR*(L2) four haplotypes (AC, AT, GC, GT) and milk yield and quality traits.

Traits	AC (n = 90)	AT (n = 92)	GC (n = 90)	GT (n = 128)
Milk yield at 305 day (kg)	1856.56 ± 43 ^cC^	2026.24 ± 41 ^b^	2058.33 ± 40 ^bB^	2310.48 ± 39 ^aA^
Fat percentage	6.02 ± 0.10 ^cC^	6.54 ± 0.10 ^b^	6.468 ± 0.12 ^bB^	7.15 ± 0.14 ^aA^
Protein percentage	4.00 ± 0.07 ^cC^	4.39 ± 0.06 ^bB^	4.35 ± 0.08 ^b^	4.85 ± 0.10 ^aA^
Lactose percentage	5.17 ± 0.19	5.35 ± 0.21	5.21 ± 0.18	5.44 ± 0.16
Total solid percentage	16.62 ± 0.30	17.24 ± 0.38	17.05 ± 0.34	17.19 ± 0.36

Data are expressed as least squares means ± SEM. Different lowercase letters indicate significant differences between haplotypes (*p* < 0.05). Different uppercase letters indicate significant differences between haplotypes (*p* < 0.01).

## Data Availability

The data supporting the present findings are contained within the manuscript.

## Supplementary Materials

The following are available online at https://www.mdpi.com/article/10.3390/ani11051237/s1, Figure S1: Agarose gel shows ampilified PRLR(L1) fragments (212bp) in 7 different buffaloes, Figure S2: PCR-SSCP bands of the PRLR(L1) in 5 buffaloes show similar pattern, Figure S3: Sequence chromatogram of PRLR(L1) shows exon 3, partial sequence of intron 2 and 3, coding sequences (CDS), amino acid sequences (colored letters above the yellow bar), and forward and reverse primers, Figure S4: The sequences of the different 5 SSCP patterns (P1-P5) and the combined genotypes of PRLR(L2) in Egyptian river buffalo, Figure S5: A comparison between sequence of PRLR(L1) in Egyptian river buffalo (this study) and both Indian river buffalo (GenBank accession number GQ339914) and Italian river buffalo (GenBank accession number MF461277.1) showed an insertion mutation (the first gap in the green bar) in non-coding sequence of E3 and g.1268G>T (p. Val19Phe) SNP (the second gap) among Egyptian and foreign buffaloes, Figure.S6: Pair-wise linkage disequilibrium (LD) analysis revealed a very strong linkage disequilibrium (D’= 1, as indicated by red diamond one block) between g.11685G>A (c.1480 G>A) and 1.98 in g.11773T>C (c.1568 T>C) SNP, Table S1: Dilutions and sources of antibodies used in western blot, Table S2: Comparative analysis of SNPs detected in E10 of PRLR(L2) between Egyptian water buffalo (this study) and the GenBank published sequences of various ruminant species.
