# Supplementary material for: Polymorphisms of the PRLR Gene and Their Association with Milk Production Traits in Egyptian Buffaloes
Source: Animals (Basel). 2021 Apr 25;11(5):1237. doi: 10.3390/ani11051237 (PMC8146870; doi:10.3390/ani11051237)
Supplement: Supplementary file 1 [file animals-11-01237-s001.zip › animals-1165269-supplementary.pdf]

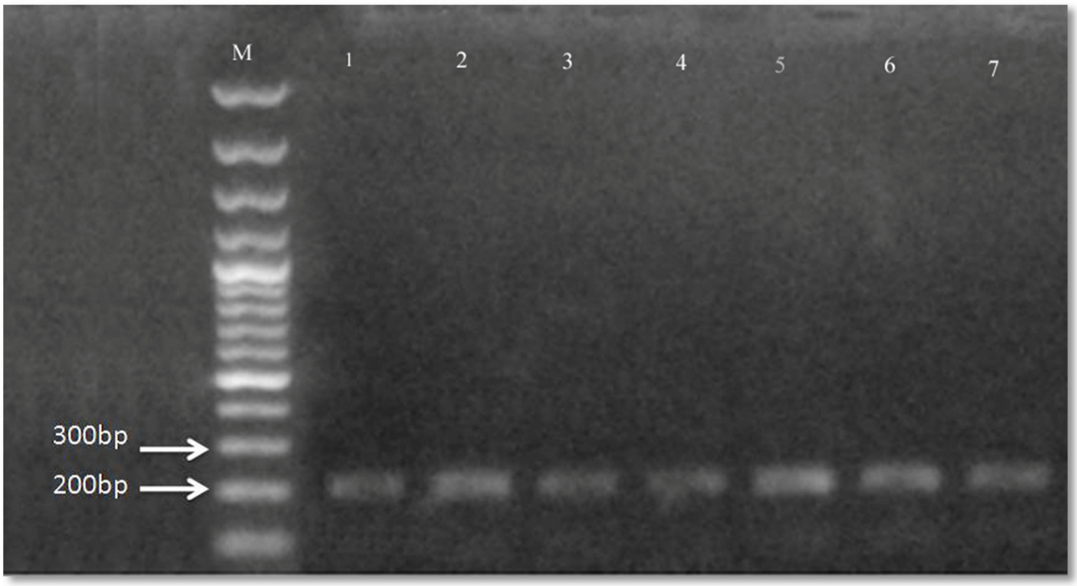

Fig.S1. Agarose gel shows ampilified *PRLR*(L1) fragments (212bp) in 7 different buffaloes**.** M represents 100 bp DNA marker.

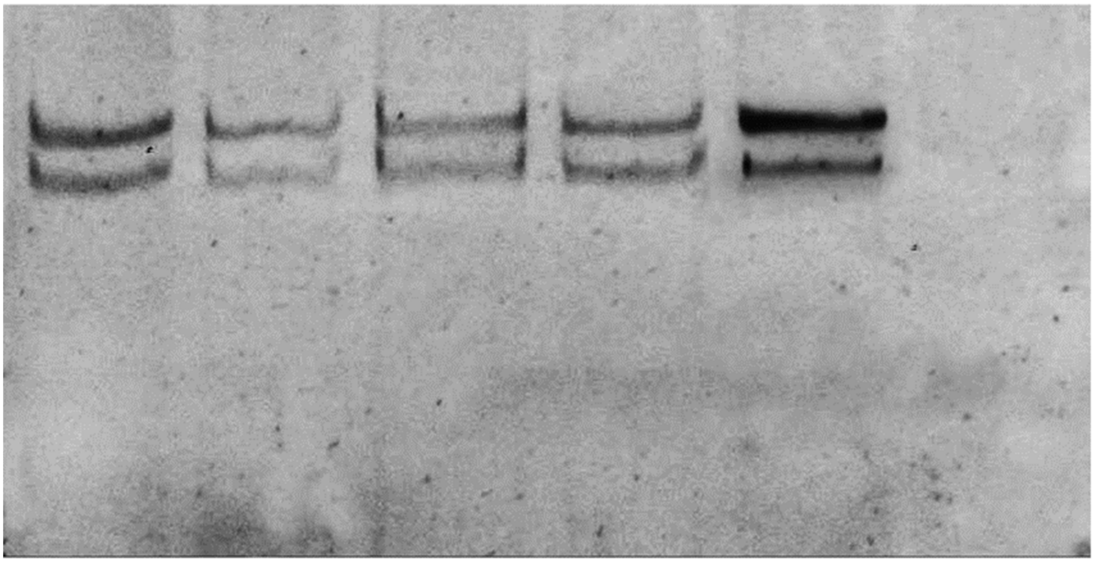

Fig.S2. PCR-SSCP bands of the *PRLR*(L1) in 5 buffaloes show similar pattern.

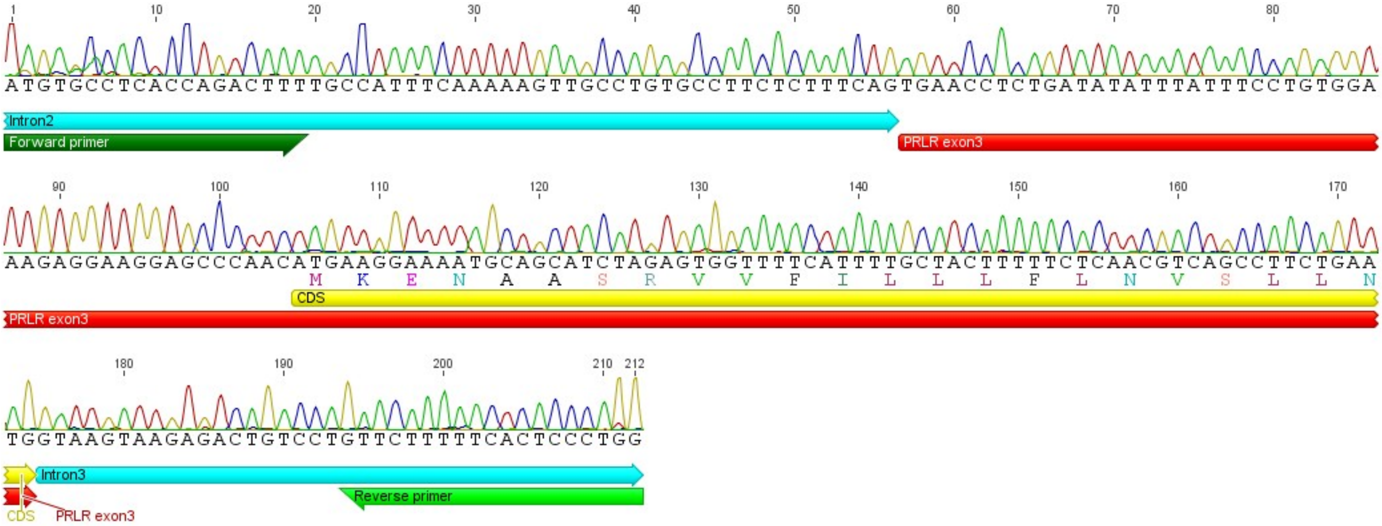

Fig.S3. Sequence chromatogram of *PRLR*(L1) shows exon 3, partial sequence of intron 2 and 3, coding sequences (CDS), amino acid sequences (colored letters above the yellow bar), and forward and reverse primers.

P1 (GA/TC)

TGGACCAAACAGACCAACATGCTTTAAAAGCCTCAAAAACCATTGAGACTGGCA
GGGAAGGAAAGGCAACCAAGCAGAGTGAGTCAGAAGGCTACAGTTCCAAGCCTG
ACCAAGACACG(G/A)CGTGGCCACTACCCCAAGACAAAACCCCCTTGATCTCTGC
TAAACCCTTGGAATATGTGGAGATCCACAAGGTCAGCCAAGATGGAG(T/C)GCTG
GCTCTGTTCCCAAAACAAAACGAGAAGGTTGGCGCCCCTGAAACCAGCAAGGAG
TACTCAAAGGTGTCCCAGGTGACAGATAGCAACATCCTG

P2 (GG/TT)

TGGACCAAACAGACCAACATGCTTTAAAAGCCTCAAAAACCATTGAGACTGGCA
GGGAAGGAAAGGCAACCAAGCAGAGTGAGTCAGAAGGCTACAGTTCCAAGCCTG
ACCAAGACACGGCGTGGCCACTACCCCAAGACAAAACCCCCTTGATCTCTGCTAA
ACCCTTGGAATATGTGGAGATCCACAAGGTCAGCCAAGATGGAGTGCTGGCTCTG
TTCCCAAAACAAAACGAGAAGGTTGGCGCCCCTGAAACCAGCAAGGAGTACTCA
AAGGTGTCCCAGGTGACAGATAGCAACATCCTG

P3 (GG/TC)

TGGACCAAACAGACCAACATGCTTTAAAAGCCTCAAAAACCATTGAGACTGGCA
GGGAAGGAAAGGCAACCAAGCAGAGTGAGTCAGAAGGCTACAGTTCCAAGCCTG
ACCAAGACACGGCGTGGCCACTACCCCAAGACAAAACCCCCTTGATCTCTGCTAA
ACCCTTGGAATATGTGGAGATCCACAAGGTCAGCCAAGATGGAG(T/C)GCTGGCT
CTGTTCCCAAAACAAAACGAGAAGGTTGGCGCCCCTGAAACCAGCAAGGAGTAC
TCAAAGGTGTCCCAGGTGACAGATAGCAACATCCTG

P4 (AA/CC)

TGGACCAAACAGACCAACATGCTTTAAAAGCCTCAAAAACCATTGAGACTGGCA
GGGAAGGAAAGGCAACCAAGCAGAGTGAGTCAGAAGGCTACAGTTCCAAGCCTG
ACCAAGACACGACGTGGCCACTACCCCAAGACAAAACCCCCTTGATCTCTGCTAA
ACCCTTGGAATATGTGGAGATCCACAAGGTCAGCCAAGATGGAGCGCTGGCTCT
GTTCCCAAAACAAAACGAGAAGGTTGGCGCCCCTGAAACCAGCAAGGAGTACTC
AAAGGTGTCCCAGGTGACAGATAGCAACATCCTG

P5 (GA/TT)

TGGACCAAACAGACCAACATGCTTTAAAAGCCTCAAAAACCATTGAGACTGGCA
GGGAAGGAAAGGCAACCAAGCAGAGTGAGTCAGAAGGCTACAGTTCCAAGCCTG
ACCAAGACACG(G/A)CGTGGCCACTACCCCAAGACAAAACCCCCTTGATCTCTGC
TAAACCCTTGGAATATGTGGAGATCCACAAGGTCAGCCAAGATGGAGTGCTGGC
TCTGTTCCCAAAACAAAACGAGAAGGTTGGCGCCCCTGAAACCAGCAAGGAGTA
CTCAAAGGTGTCCCAGGTGACAGATAGCAACATCCTG

Fig.S4. The sequences of the different 5 SSCP patterns (P1-P5) and the combined genotypes of PRLR(L2) in Egyptian river buffalo. Red colored nuecleotides are the SNPs.

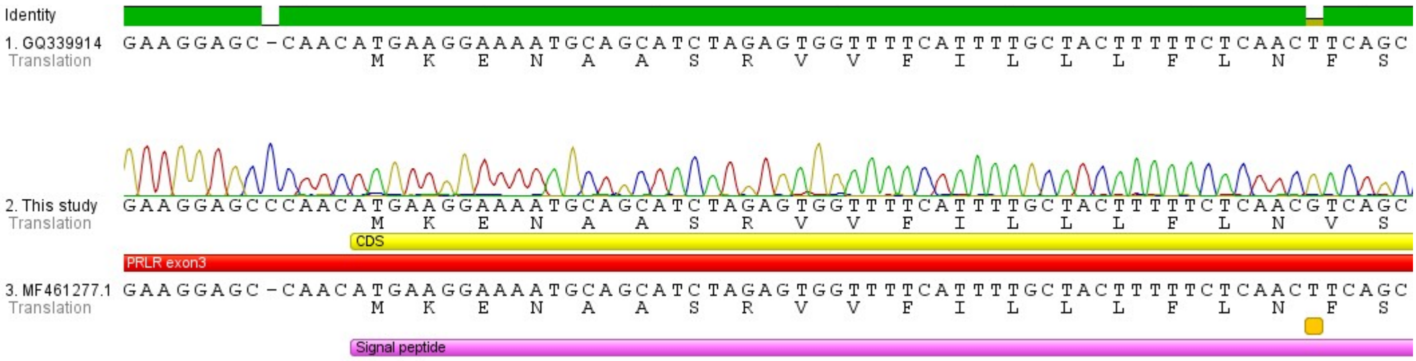

Fig.S5. A comparison between sequence of *PRLR*(L1) in Egyptian river buffalo (this study) and both Indian river buffalo (GenBank accession number GQ339914) and Italian river buffalo (GenBank accession number MF461277.1) showed an insertion mutation (the first gap in the green bar) in non-coding sequence of E3 and g.1268G>T (p. Val19Phe) SNP (the second gap) among Egyptian and foreign buffaloes. The violet bar shows the signal peptide domain of *PRLR*.

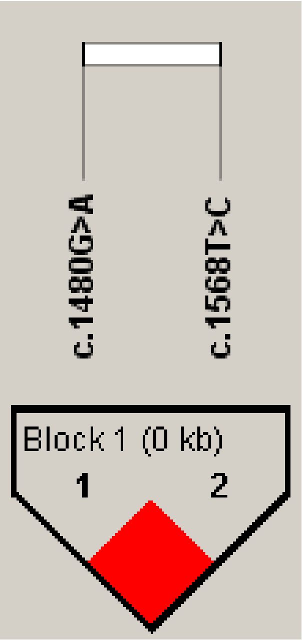

Fig.S6. Pair-wise linkage disequilibrium (LD) analysis revealed a very strong linkage disequilibrium (D'= 1, as indicated by red diamond one block) between g.11685G>A (c.1480 G>A) and 1.98 in g.11773T>C (c.1568 T>C) SNP.

Table S1: Dilutions and sources of antibodies used in western blot.

	Name	Dilution	Size (KDa)	Source	Catalogue number
4	Anti-β-actin primary ab	1:200	43.3	Santa Cruz Biotechnology, Inc., USA	sc-47778
5	Bovine anti-PRL polyclonal primary ab	1:300	22.7	Cloud-Clone Corp., USA	PAA846Bo01
6	Bovine anti-PRLR primary ab	1:300	28	MyBioSource, San Diego, USA	MBS2026486
8	Horseradish peroxidase conjugated anti-rabbit IgG secondary ab	1:5000		Santa Cruz Biotechnology, Inc., USA	sc-2030

Table S2. Comparative analysis of SNPs detected in E10 of *PRLR*(L2) between Egyptian water buffalo (this study) and the GenBank published sequences of various ruminant species.

locus*	PRLR(L2) (E10 from nt g.11566 to g.11870)													
nt position	11577		11580		11683		11685		11687		11768		11773	
SNP	G>A		A>C		C>T		G>A		A>G		T>C		T>C	
aa position	458		459		493		494		494		521		523	
aa change	Asp/Asn		Lys/Gln		Thr/Met		Ala/Thr		Ala		Asp		Val/Ala	
Codon change	GAC	**A**AC	AAA	**C**AA	ACG	A**T**G	GCG	**A**CG	GCA	GC**G**	GAT	GA**C**	GTG	G**C**G
Egyptian Bubalus bubalis[1]	GAC		CAA		ACG		GCG	**A**CG	GCG (Ala)	**A**CG (Thr)	GAT		GTG	G**C**G
Italian Bubalus bubalis[2]	GAC	**A**AC	AAA	**C**AA	ACG	A**T**G	GCG		GCA	GC**G**	GAT	GA**C**	GTG	
Indian Bubalus bubalis[3]	GAC		AAA	**C**AA	ACG		GCG		GCA	GC**G**	GAT		GTG	
Bos taurus[4]	GAC		CAA		ACG		GTG (Val)		GTG (Val)		GAT		GTG	
Capra hircus[5]	GAC		CAA		ATG		GCA(Ala)		GCA		GAT		GTA(Ala)	
Ovis aries[6]	GAC		CAA		ATG		GCA(Ala)		GCA		GAT		GTA(Ala)	
Camelus dromedarius[7]	GAC		AAA		ACG		GTG (Val)		GTG (Val)		GAT		GCG	

* SNP position was determined based on the published Italian river buffalo sequence (accession no MF461277.1). Blue color nucleotides refer to mutant alleles. aa, amino acid; E10, exon10; nt, nucleotide.

[1]This study; [2]GenBank accession number MF461277.1; [3] HQ236497 and GQ339914; [4]L02549.1; [5]KJ792813.1; [6]AF041257; [7]XM_010977605.
